# Notes and Notices

**Published:** 1895-09

**Authors:** 


### NOTES AND NOTICES.

DR. F. E. BŒRICKE, senior member of the firm of Bœricke & Tafel, has been appointed by the Governor of Pennsylvania a member of the State Pharmaceutical Examining Board of Pennsylvania, as a representative of Homœopathic Pharmacy.

THE HOMŒOPATHIC MEDICAL SOCIETY of the State of New York will hold its forty-fourth semi-annual meeting in New York city, October 1st and 2d, 1895, and its forty-fifth annual meeting at Albany, February 11th and 12th, 1895. President, Charles E. Jones; Vice-Presidents, W. B. Glifford, D. J. Roberts, and W. Louis Hartman; Treasurer, Charles Deady, and Secretary John L. Moffat, M. D., 17 Schermerhorn Street, Brooklyn, N. Y.

DR. FRANK PARSONS NORBURY, who recently removed to St. Louis to assume the editorial management of the *Medical Fortnightly*, has been elected to the chair of Pratice of Medicine and Clinical Medicine in the St. Louis College of Physicians and Surgeons.

VACCINATION.—I desire deliberately and publicly to repeat that I regard every child who dies of compulsory vaccination to be murdered.—EMERITUS PROFESSOR FRANCIS W. NEWMAN.

